# Chronic traumatic encephalopathy (CTE) in the context of longstanding intimate partner violence

**DOI:** 10.1007/s00401-024-02757-3

**Published:** 2024-07-08

**Authors:** M. Tiemensma, R. W. Byard, R. Vink, A. J. Affleck, P. Blumbergs, M. E. Buckland

**Affiliations:** 1 Forensic Pathology Unit, Royal Darwin Hospital, Darwin, NT Australia; 2 College of Medicine and Public Health, Flinders University, Adelaide, Australia; 3 The University of Adelaide, Frome Rd, Adelaide, 5005 Australia; 4 Forensic Science SA, 21 Divett Place, Adelaide, 5000 Australia; 5 Clinical and Health Sciences, University of South Australia, Adelaide, 5001 Australia; 6 Brain and Mind Centre, University of Sydney, Camperdown, NSW Australia; 7 Department of Neuropathology, Royal Prince Alfred Hospital, Camperdown, NSW Australia

Chronic traumatic encephalopathy (CTE) is defined by the abnormal accumulation of hyperphosphorylated tau (p-tau) in neurons around blood vessels at the depths of cortical sulci. CTE is found almost exclusively in people with a history of repeated mild traumatic brain injury [1]. While the majority of CTE cases have been reported in males playing contact sports, anyone experiencing repetitive head injury (RHI) is potentially at risk [2].

Intimate partner violence (IPV) is a global public health issue, with most recent estimates indicating that nearly 1 in 3 or 736 million women have experienced IPV [7]. CTE was first linked with IPV in 1990 [6], where the deceased had suffered physical abuse for ‘many years’. Evidence of RHI was found in the form of ‘abnormal thickening of the ears, resembling “cauliflower ears” of pugilists’ [6]. In 2021, another case was published using contemporary CTE diagnostic criteria and occurring on a background of years of abuse with “cauliflower ears” and numerous scars of the scalp [5]. Here we report two additional cases of CTE in the context of IPV.

Forensic autopsies were performed on two women with significant vulnerability, complex health issues, and RHI in the context of longstanding IPV (Table 1). Multiple facial and scalp scars were present in both cases, as well as recent scalp lacerations. There was no evidence of recent or remote strangulation/neck compression. Screening for CTE was based on current recommendations [1].

**Table 1:** Case information

	Case 1	Case 2
Age (decade)	5th	4th
Clinical history		
Hazardous alcohol use	** + **	** + **
Diabetes mellitus	** + **(Type 2)	** + **(Type 3c)
Rheumatic heart disease	+	–
Hypertension	+	–
Dyslipidemia	** + **	–
Chronic pancreatitis	**–**	+
Chronic electrolyte disturbances	**–**	+
Cognitive Impairment (RUDAS score)	No (25/30)	Possible (21/30)
Intimate partner violence history		
No. of IPV years	20 +	17
No. of assault-related medical presentations	30 +	40 +
No. of recorded head injuries^a^	15 +	20 +
Pathological findings		
CTE stage	Low (McKee II)	Low (McKee I)
ADNC	“Low” (A1, B1, C0)	–
PART	–	Possible (Braak stage I)
ARTAG	Subpial	–
Microinfarcts	Cortical, old	–
SVD	Mild	Mild
CAA	+	–
Traumatic axonal injury	Diffuse	–
Cause of death	Blunt force injuries (alleged assault)	Blunt impact trauma (struck by motor vehicle)

*ADNC* Alzheimer disease neuropathologic changes, *ARTAG* aging-related tau astrogliopathy, *CAA* cerebral amyloid angiopathy, *CTE* chronic traumatic encephalopathy, *IPV* intimate partner violence, *PART* primary age-related tauopathy, *RUDAS* Rowland Universal Dementia Assessment Scale, *SVD* small vessel disease

^a^Separate medically documented events ranging from facial and scalp lacerations to facial fractures (all related to IPV)

Case 1 showed smears of fresh subarachnoid blood over frontoparietal and left occipital regions, with histological evidence of old subdural hemorrhage. Six old microinfarcts were seen in frontal lobes. Mild hyaline arteriolosclerosis was present in basal ganglia. Scattered white matter beta-APP-positive varicosities were present, indicative of diffuse traumatic axonal injury. Four perivascular foci of neuronal p-tau immunoreactivity were present at sulcal depths, diagnostic for CTE [1]. Each measured ~ 2 mm in diameter, located in inferior parietal lobule, ventrolateral frontal cortex, and anterior temporal lobes (Fig. 1a–c). Three non-sulcal perivascular foci of neuronal p-tau were also present. Subpial thorn-shaped astrocytes were seen in anterior cingulate cortex and pons, indicative of low-level aging-related tau astrogliopathy (ARTAG). Low-level Alzheimer disease neuropathologic changes were seen (A1, B1, C0), with mild cerebral amyloid angiopathy in leptomeninges and neocortex.

**Fig. 1:**
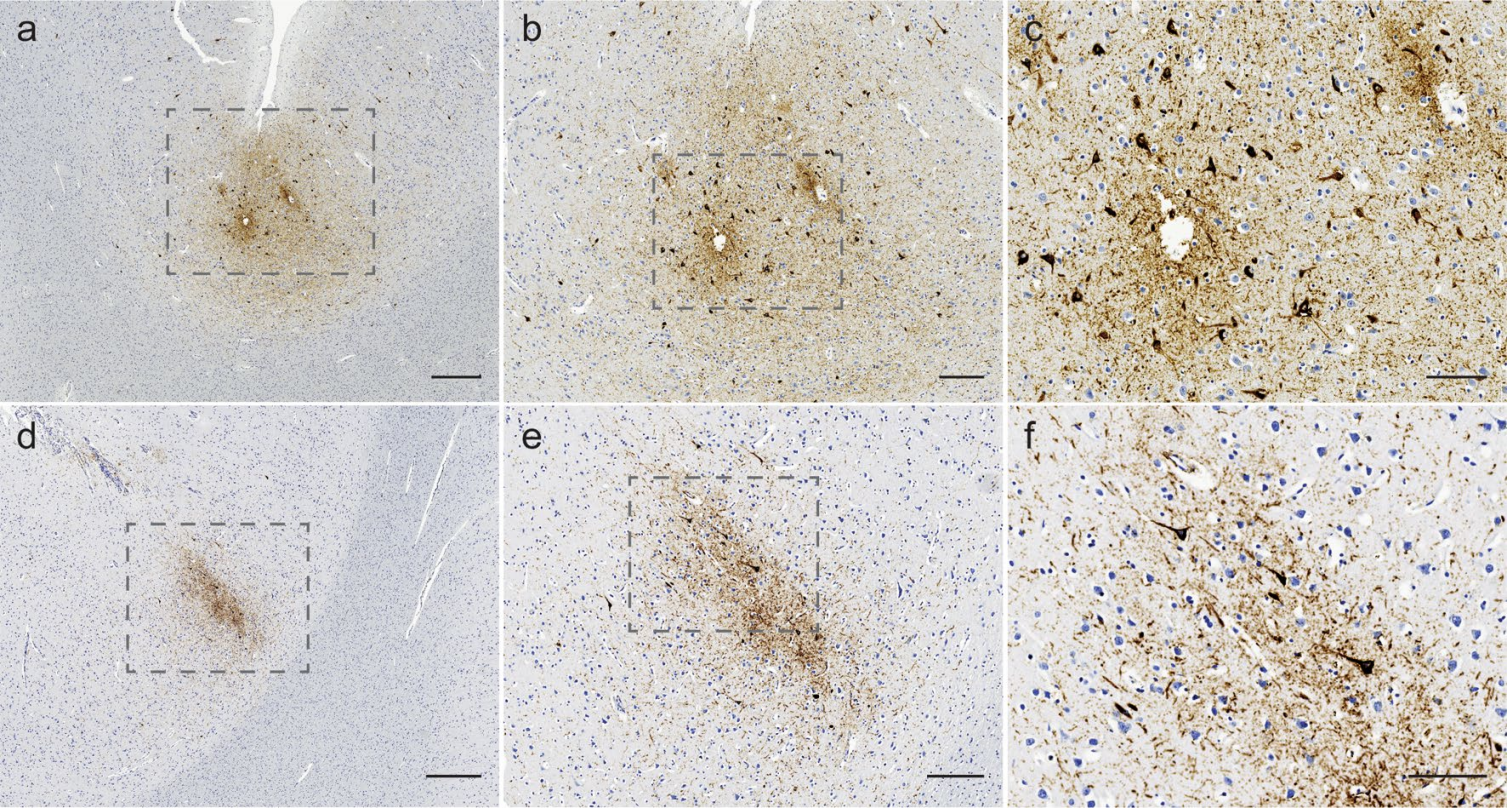
Representative CTE lesions from case 1 (**a–c**) and case 2 (**d–f**) visualized by p-tau (AT8) immunostaining. The dotted box details region of interest magnified in adjacent panel. Scale bars: **a**, **d** = 500 µm, **b**, **e** = 200 µm and **c**, **f** = 100 µm

The brain of Case 2 was macroscopically unremarkable. Histological examination identified mild gliosis and mild hyaline arteriolosclerosis of subcortical white matter. Immunohistochemistry revealed a 2–3 mm perivascular focus of neuronal p-tau in left dorsolateral frontal cortex fulfilling CTE diagnostic criteria (Fig. 1d–f) [1]. Four additional regions showed low-level neuronal p-tau foci at sulcal depths without obvious perivascular arrangement. There was rare neuronal p-tau staining in transentorhinal cortex, and scant neuritic staining in entorhinal cortex, CA1, and subiculum. This may represent co-existent primary aging-related tauopathy (PART), Braak stage I, although current PART criteria exclude this diagnosis in the presence of other tauopathies [3]. Immunohistochemistry for beta-A4, alpha-synuclein and TDP-43 was negative.

In common with almost all other cases of CTE, the cases presented here had a long history of RHI. In contrast, a recent study of IPV neuropathology failed to identify CTE in a series of prospective and retrospective cases [4]. Evidence of longstanding RHI was lacking in this detailed study but has been present in all cases of CTE identified in IPV to date, underscoring the importance of chronic RHI exposure in CTE pathogenesis. As CTE is typically associated with cognitive and behavioral symptoms, future IPV interventions need to recognize the possibility of these deficits affecting individuals with longstanding RHI exposure, with intensive and specialized support for those at risk.

## Data Availability

The data that support the findings of this study are not openly available due to reasons of sensitivity, but reasonable requests for data access will be considered by the authors.
